# Navigating multidimensional obstacles: a comparison between young and older adults and among obstacles

**DOI:** 10.1371/journal.pone.0338968

**Published:** 2026-02-20

**Authors:** Ashlyn M. Jendro, Hope M. Looper, Tiphanie E. Raffegeau, Abigail C. Schmitt

**Affiliations:** 1 Department of Health, Human Performance, and Recreation, University of Arkansas, Fayetteville, Arkansas, United States of America; 2 School of Kinesiology, George Mason University, Manassas, Virginia, United States of America; 3 Department of Orthopaedics, University of Arkansas for Medical Sciences, Little Rock, Arkansas, United States of America; Drexel University School of Biomedical Engineering Science and Health Systems, UNITED STATES OF AMERICA

## Abstract

Most falls in older adults are due to tripping over objects. Older adults cross obstacles with greater toe clearance to prevent tripping, indicating obstacles may be more consequential when compared to young adults. The typical dowel rod used in obstacle crossing research is foreign and sometimes unrealistic compared to real-world obstacles. This study compared measures of obstacle crossing between young and older adults using four different obstacles. Thirty young (23 ± 4 years) and 30 older adults (69 ± 6 years) crossed four different obstacles: a branch, a parking curb, a dowel rod, and a puddle. Motion capture data was used to compute measures of toe clearance, approach/landing distances, and lower body joint kinematics. We analyzed different combinations of obstacles within the older adult group and between groups for each obstacle using analysis of variance tests with False Discovery Rate corrections applied to *post-hoc* comparisons. A total of 1196 young adult and 1233 older adult obstacle crossing trials were analyzed. Older individuals had greater clearance in leading toe vertical clearance across all obstacles, but only among the branch and dowel for trailing toe vertical clearance. The dowel produced the greatest leading toe vertical clearance in both groups. No differences were observed in approach/landing distances, crossing step length, or obstacle crossing speeds. Older adults crossed all obstacles with greater peak ankle dorsiflexion on the lead limb, and more trail limb peak ankle dorsiflexion for the branch and curb. Older adults exhibited higher toe clearance in both leading and trailing limbs during crossing compared to younger adults, likely from greater ankle dorsiflexion. By increasing toe clearance, older adults increase the margin of safety, decreasing the likelihood of contacting the obstacle and potentially falling. Collectively, these results better our understanding of how realistic obstacle crossing changes with age across different obstacles.

## Introduction

In 2020, three million emergency department visits were due to falls among older adults, resulting in approximately $50 billion in medical costs [1]. Falls among older adults can be devastating, as they can cause injuries that may reduce the ability to remain independent. The majority of falls are caused by body imbalance or obstacle collision, and falls are considered a leading cause of injury and death among older individuals [2–4]. Dysfunction within the locomotor system can lead to imbalances and instability in older adults, thus predisposing them to trip over obstacles [5]. The majority of falls in older adults are due to tripping over a clearly visible stationary object (e.g., rug, chair, bag) [6–8]. When obstacle crossing, visual information is critical for crossing with the lead limb (the limb that crosses over the obstacle first). However, we rely on *remembered* visual information to make successful crossings for the trailing limb (the limb that crosses over the obstacle second) since the obstacle is outside the lower visual field after the lead limb successfully crosses [9–11].

To cross obstacles, older individuals use greater hip flexion and hip adduction in the lead limb to clear the obstacle and increased ankle dorsiflexion in the trailing limb to stabilize themselves [12]. Older adults are also more conservative when approaching obstacles, as observed by decreased approach speeds, crossing speeds, and step lengths [13,2]. To account for decreased muscle strength due to aging, older individuals reduce the anteroposterior distance between their center of mass (COM) and center of pressure (COP) to decrease joint loading during single support [2]. The decrease in COM-COP separation promotes an increased ability to recover in the case of a collision by allowing extra time to correct foot placement [14–16]. This conservative strategy decreases the mechanical load that challenges older adults [2] and reduces the likelihood of a fall [2,17]. Likewise, older adults cross obstacles with increased toe clearance to prevent tripping and remain safe during obstacle crossing, suggesting they perceive the obstacle as a greater challenge with more severe consequences compared to healthy young individuals [12].

Historically, a dowel rod is used as an obstacle in obstacle crossing literature that has informed fall prevention strategies and education [10,13,18,19]. Although the dowel rod system is inexpensive, easy to replicate and adjust height, as well as safe to use for participants in a research setting, the dowel is foreign and sometimes unrealistic when compared to obstacles encountered in real-world situations [20]. While the dowel may have similar dimensions to real-world obstacles such as a branch, it is a poor proxy for obstacles with depth such as a parking curb or a puddle [20]. To our knowledge, it is currently unknown how older adults cross obstacles with significant depth.

The purpose of this study was to compare measures of obstacle crossing behavior between young and older adults using four different real-world or simulated real-world obstacles: a branch, a parking curb, a dowel rod, and a puddle. We hypothesized that older adults will generally prioritize safety when crossing obstacles, compared to young adults, demonstrated by decreasing the approach and landing distance (distance between the foot and the obstacle) for both the lead and trail limbs, while increasing the toe clearance (vertical distance of the foot above the obstacle) to ensure successful crossings. We also hypothesized, based on previous findings [20], that among older adults, the dowel will elicit increased measures of obstacle clearance (increased approach and landing distance, as well as increased toe clearance and decreased crossing velocity) when compared to the other obstacles.

## Materials and methods

### Participants

Thirty young adults (20 women, 10 men; age: 23 ± 4 years; height: 1.66 ± 0.10 m; mass: 69.7 ± 16.5 kg; 26 right-foot dominant, 4 left-foot dominant; overground unobstructed preferred walking speed: 1.23 ± 0.12 m/s) and 30 older adults (18 women, 12 men; age: 69 ± 6 years; height: 1.67 ± 0.09 m; mass: 75.5 ± 18.1 kg; 28 right-foot dominant, 1 left-foot dominant, 1 missing due to data collection error; overground unobstructed preferred walking speed: 1.25 ± 0.17 m/s) who could ambulate unassisted for more than 15 minutes without rest participated in this study. The older adult age range (60–80 years) was chosen to target individuals that are likely experiencing age-related gait changes but are still living independently and community ambulators. Participants were excluded if they had an uncontrolled neurological or orthopedic condition or if they self-reported a recent musculoskeletal injury that could impair walking function, precluding their ability to safely participate. All participants were recruited between January 11, 2022 and October 27, 2023 and they provided written informed consent prior to participation. This study was approved by the University’s Institutional Review Board (protocol # 2105335358).

### Data acquisition

Participants completed a health history assessment, the Short Physical Performance Battery (SPPB) [21], the Montreal Cognitive Assessment (MoCA) [22] and the Trail Making Test (TMT; forms A and B) [23,24]. The health history questionnaire was used in determining study eligibility. The SPPB was used to ensure they could safely complete the obstacle crossing tasks [25]. The MoCA and TMTs were used to characterize the sample as demographic variables. Further, older adult participants were asked to rate how safe they felt crossing each obstacle prior to attempting using a five-point Likert scale, with “1” indicating “completely unsafe” and “5” indicating “completely safe”. If participants felt it was unsafe to cross an obstacle, they were allowed to skip that obstacle.

**Fig 1:**
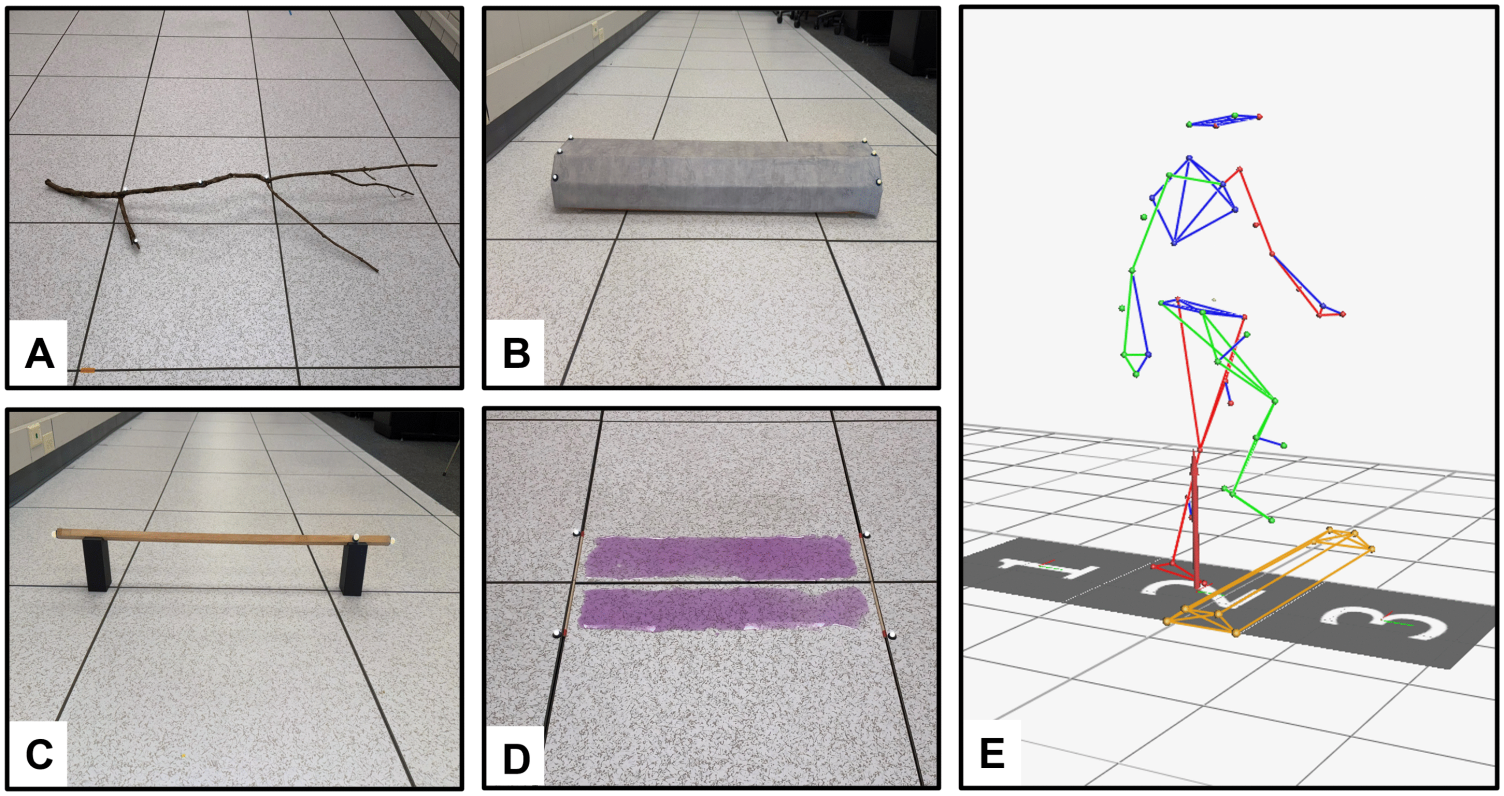
Obstacles presented in data collection. (A) branch, (B) parking curb, (C) dowel rod, and (D) puddle. Panel E illustrates a participant crossing the curb obstacle.

Participants were barefoot and fitted with the Plug-in-Gait™ full body marker set (Vicon Motion Systems, Oxford, UK) and completed 10 trials each of 5 walking conditions; an unobstructed walking condition, which served as our familiarization trials, and four obstructed walking conditions. During familiarization trials, participant start points were adjusted to ensure foot placement would naturally align on either side of the obstacles during the subsequent obstructed trials. Marker trajectories were captured using sixteen Vicon motion capture cameras (100 Hz) [6,13]. Obstructed walking conditions were presented in a randomized order and included: 1) a branch (height: 170 mm, depth: 25 mm), 2) a parking curb (height: 120 mm, depth: 215 mm), 3) a dowel rod (height: 150 mm, depth: 25 mm), and 4) a puddle (height: 2 mm, depth: 300 mm) (Fig 1). Each condition included at least 10, 8-meter overground walking trials along a walkway and participants were instructed to *“...walk across the walkway at a comfortable pace...stepping over the [obstacle name] in your path along the way”*. Participants were encouraged to walk at their preferred pace and stepped over the obstacle with their self-selected limb. Additional, standardized instructions were given for the puddle as: *“This mixture is slippery and will cause you to slip on the floor if you contact it. Please do not step in it to prevent slipping”*. Puddle composition was purple liquid glue and water to give the illusion of a slippery surface and make it easier for participants to identify, however, participants were blinded to the solution makeup. All obstacles were positioned or applied in full view of the participants.

### Data analysis

Trajectory data was processed within Nexus (Vicon Motion Systems) and MATLAB (MathWorks, Natick, MA, USA). Trajectories were filtered using a Butterworth low-pass, fourth order, zero-lag filter with a cut-off frequency set to 6 Hz prior to Plug-in-Gait model calculations for joint kinematics. Exported trajectory data was run through a custom MATLAB code to extract the dependent variables of interest. All obstacles were identified with retroreflective markers for customized tracking within Nexus (Fig 1).

**Fig 2:**
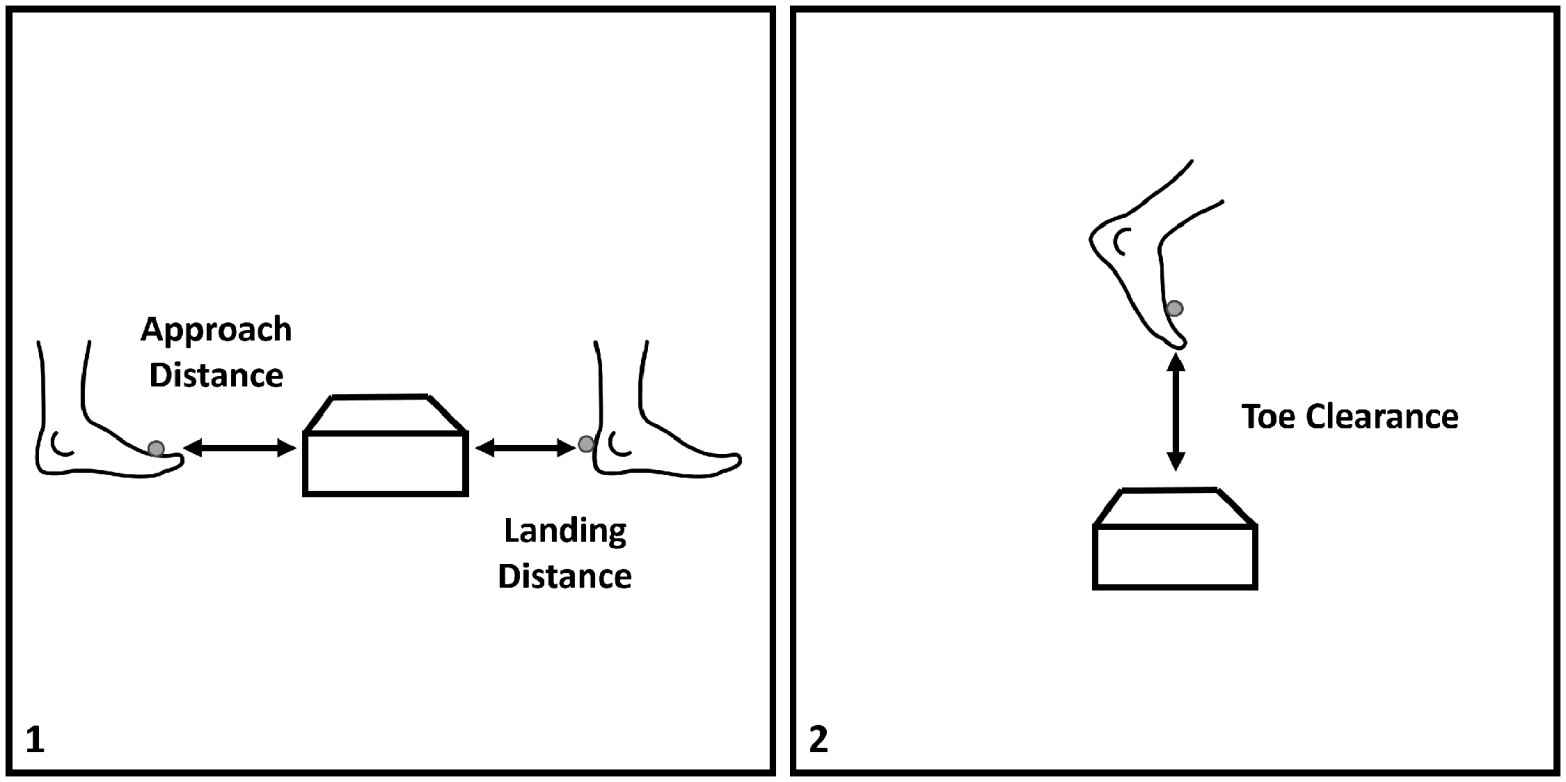
Schematics from the sagittal view to illustrate how clearance measures were assessed for (1) horizontal clearance measures (i.e., approach and landing distance), and (2) vertical toe clearance measures.

Measures of toe clearance, approach/landing distances, and lower body joint kinematics were used to assess obstacle crossing strategy. The minimum vertical toe clearance over each obstacle was measured in millimeters when the toe marker was directly over the obstacle, for both the leading and trailing legs. Approach distance was defined as the horizontal distance between the trailing limb toe marker and the leading edge of the obstacle when the leading limb was directly over the obstacle (i.e., during single support). Landing distance was defined as the horizontal distance between the leading limb heel marker and the trailing edge of the obstacle during the step over the obstacle (Fig 2). The leading edge of the obstacle is the edge closest to the participant prior to crossing, while the trailing edge is the edge of the obstacle closest to the participant after crossing.

Two spatiotemporal measures of interest were calculated: crossing velocity and crossing step length. Crossing velocity was calculated as the center of mass velocity during the crossing phase, defined as toe-off of the leading limb to heel-strike of the trailing limb. Crossing step length was calculated as the distance from the heel marker of the trailing foot to the heel marker of the contralateral leading foot, at heel-strike, when participants were over the obstacle (i.e., the step straddling the obstacle).

Trials were cropped to remove acceleration phases. Maximum lower extremity joint kinematics were extracted from the lead and trail limbs during the crossing step of obstacle crossing. Variables of interest were extracted from each trial and averaged across trials within each obstacle condition for each participant. Unsuccessful trials, which we defined as contact with the obstacle, were removed from analysis along with the subsequent trial. The subsequent trials were removed due to the likely inflated measures attributable to motor learning error corrections after obstacle contact [26–29].

### Statistical analysis

Dependent variables were checked for normality and sphericity using Shapiro-Wilks test and Mauchly’s tests, respectively. Since the primary goal of this study was to compare young and older adults while also examining the differences in crossing strategies between obstacles, we decided *a priori* to examine the interaction between the effect of aging (group) and obstacle, but not the interaction between every linear combination of group and obstacle. Young adult data for obstacle crossing can be found at Jendro et al., 2025 [20]. The focus of this analysis is between the different combinations of obstacles within the older adult group and between groups for each obstacle type. For example, we did not compare differences between older adults crossing the dowel with younger adults crossing the puddle. Therefore, we used a combination of 2 x 4 analysis of variance (ANOVAs) to compare means of the dependent variables between age and conditions and a series of linear mixed effects regression models (*lmer*) to analyze the relationship between age (group) and condition (obstacle). If the ANOVA resulted in significant differences (*p* < .05), False Discovery Rate (FDR) corrections were applied to the *post-hoc* comparisons (*emmeans*) for the interaction (α = .05, number of comparisons = 10) or the main effect of obstacle (α = .05, number of comparisons = 6) [30,31]. Finally, Cohen’s *d* was used to characterize the magnitude of the effect between pairwise comparisons (*eff_size* with summed variance components). Effect size was interpreted as small (0.2 ≤ *d* < 0.5), medium (0.5 ≤ *d* < 0.8) or large (*d* ≥ 0.8) [32]. Statistical analyses were completed using R (v. 4.4.0 “Puppy Cup”) [33] and RStudio (v. 2024.04.1 + 748 “Chocolate Cosmos”).

## Results

In the young adult group, a total of 3 crossing trials were unsuccessful, including 2 from the curb, and 1 from the puddle. A total of 7 crossing trials were unsuccessful in the older adult group, including, 1 from the branch, 2 from the curb, and 4 from the puddle, however, six of the seven contacts were from a single participant. A total of six trials from the young adult group and 13 trials from the older adult group were removed from the analysis as either unsuccessful crossings or the subsequent trial (i.e., as one older adult participant had two obstacle contacts with the puddle in a row, only the trial after the last contact was removed.). After removal, 1196 young adult trials and 1233 older adult trials remained for analysis. Older adults crossed the obstacles with their self-reported dominant leg 49% of the time (range: 0% to 100%) whereas young individuals crossed the obstacles with their self-reported dominant leg 40% of the time (range: 0% to 100%). Table 1 presents information on participant falls, and SPPB, MoCA, and TMT scores.

**Table 1:** Demographic information of participants.

	Young Adults *n* = 30	Older Adults *n* = 30
**Unobstructed, preferred walking speed (m/s)**	1.23 ± 0.12	1.25 ± 0.17
**Falls in the last 6 months**		
Range	0 - 1	0 - 5
Mean ± SD	0.1 ± 0.3	0.4 ± 1.1
**SPPB score**		
Range	11 - 12	9 - 12
Mean ± SD	12.0 ± 0.2	11.7 ± 0.7
**MOCA score**		
Range	23 - 30	21 - 30
Mean ± SD	27.1 ± 2.1	27.1 ± 2.3
**Trail Making Test A**		
Time (s)	64.8 ± 16.9	85.2 ± 26.7
Errors	0.3 ± 0.8	0.9 ± 1.3
**Trail Making Test B**		
Time (s)	83.6 ± 30.4	121.8 ± 36.4
Errors	0.3 ± 0.7	1.1 ± 1.5

*Notes*: m/s = meters per second, SD = standard deviation, s = seconds, SPPB = Short Physical Performance Battery, MOCA = Montreal Cognitive Assessment. SPPB scores can range from 0–12, with greater scores indicating better lower extremity function. Normative data for the SPPB from community-dwelling older adults indicate an average score of 8.3 ± 2.7 [34]. The MOCA is scored out of 30 with higher scores indicating better cognitive function.

Twenty-eight of 30 older adults completed safety ratings for the obstacles. Scores for the branch, curb, and puddle ranged from 4–5 on the Likert scale, and the dowel ranged from 3–5, with “5” indicating participants felt “completely safe” crossing the obstacle. One older adult participant opted out of the puddle crossing after answering *“no”* to the question *“would you feel safe crossing a puddle one foot wide?”.*

### Vertical clearance measures of obstacle crossing

When considering the leading toe vertical clearance, there was not a significant interaction (F(3,178.70)= 1.76, *p* = .156), but older individuals crossed the obstacles with greater leading toe vertical clearance compared to younger adults (F(1,59.62)= 5.74, *p* = .020, *d* = 0.52) and the leading toe vertical clearance differed significantly among the obstacles (F(3,178.70)= 192.84, *p* < .001). All obstacles differed significantly from one another and displayed medium to large effects, with the greatest leading toe clearance for the dowel, followed by the branch, then the curb, and smallest for the puddle (all *p* ≤ .001, *d* range: 0.50–2.68; Table 2).

**Fig 3:**
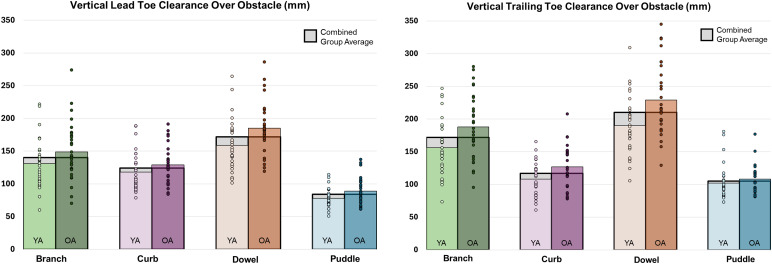
Vertical toe clearance measures (mm) when the toe was directly over the obstacle for young adults (YA) and older adults (OA).

A significant interaction between group and obstacle for trailing toe vertical clearance (F(3,179.20)= 3.90, *p* = .010) revealed differences between older and younger adults while crossing the branch and the dowel obstacles (*p* = .040, *d* = 0.85, *p* = .006, *d* = 1.00; Fig 3), but not the curb (*p* = .596, *d* = 0.49) or puddle (*p* = .999, *d* = 0.16). Further, for older adults, the trailing toe vertical clearance was greatest for the dowel compared to all other obstacles (*p* ≤ .001, *d* range: 1.06–3.14), followed by the branch compared to all other obstacles (*p* < .001, *d* range: 1.60–2.07). There were no significant differences in trailing toe vertical clearance between the curb and the puddle (*p* = .251, *d* = 0.48).

**Table 2:** Mean ± SD of measures of obstacle crossing in mm for each group.

		Lead Toe Clearance	Trail Toe Clearance	Approach Distance (TL)	Landing Distance (LL)	Crossing Step Length
Younger Adults	Branch	131 ± 37	156 ± 44	257 ± 60	239 ± 50	701 ± 70
	Curb	118 ± 30	108 ± 26	182 ± 53	145 ± 39	742 ± 67
	Dowel	159 ± 39	190 ± 44	259 ± 58	241 ± 50	709 ± 69
	Puddle	78 ± 15	102 ± 27	172 ± 50	123 ± 40	779 ± 62
Older Adults	Branch	149 ± 43	188 ± 50	249 ± 69	215 ± 64	672 ± 98
	Curb	129 ± 28	127 ± 32	171 ± 47	121 ± 59	709 ± 89
	Dowel	185 ± 39	229 ± 54	246 ± 68	208 ± 71	662 ± 102
	Puddle	89 ± 20	108 ± 22	168 ± 47	119 ± 46	768 ± 83

### Horizontal clearance measures of obstacle crossing

**Fig 4:**
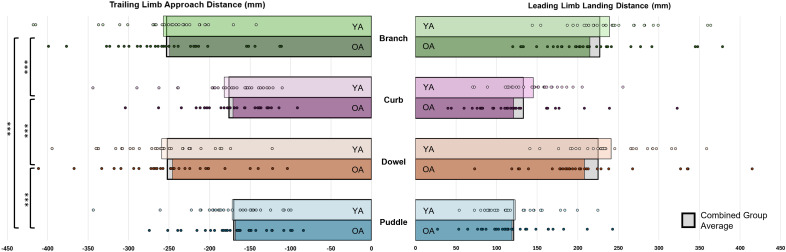
Horizontal clearance measures (mm) when the contralateral toe was directly over the obstacle. Significance indicators show main effects for obstacle (*** *p* < .001).

Although there were no interaction (F(3,179.03)= 0.524, *p* = .667) or group differences (F(1,60)= 0.392, *p* = .534) observed for the trailing limb approach distance, a main effect of obstacle (F(3,179.03)= 195.237, *p* < .001) revealed that both the branch and the dowel had longer approach distances than the curb and the puddle (*p* ≤ .001, *d* range: 1.35–1.45), but there were no differences between the branch and the dowel (*p* = .999, *d* < 0.01) or the curb and the puddle (*p* = .822, *d* = 0.09) (Table 2, Fig 4).

For leading limb landing distance, we did observe a significant interaction (F(3,179.00)= 2.91, *p* = .036), although there were no differences observed between older and younger adults after crossing the branch, (*p* = .892, *d* = 0.46), curb (*p* > .999, *d* = 0.47), dowel (*p* = .453, *d* = 0.64), or the puddle (*p* > .999, *d* = 0.11). The interaction was driven by longer landing distances for older adults when crossing the branch or the dowel compared to the curb and the puddle (*p* ≤ .001, *d* range: 1.65–1.84). Again, there were no differences between how older adults landed after crossing the branch and the dowel (*p* > .999, *d* = 0.13) or the curb and the puddle (*p* > .999, *d* = 0.07).

### Spatiotemporal measures of obstacle crossing

We observed a significant interaction for crossing step length (F(3,179.03)= 5.30, *p* = .002), driven by differences among obstacles for the older adults. There were no differences in crossing step length between older adults and younger adults for the branch, (*p* > .999, *d* = 0.36), curb (*p* > .999, *d* = 0.40), dowel (*p* = .835, *d* = 0.59), or puddle (*p* = .987, *d* = 0.15). Older adults exhibited the longest crossing step length over the puddle compared to all other obstacles (*p* ≤ .001, *d* range: 0.72–1.31) and the curb had a longer step length than either the branch (*p* < .001, *d* = 0.46) or the dowel (*p* < .001, *d* = 0.59). There was no difference between the branch and the dowel (*p* > .999, *d* = 0.13).

A significant interaction (F(3,179.02)= 3.80, *p* = .011) revealed no differences in crossing velocity between older and younger adults for the branch, (p > .999, *d* = 0.39), curb (p > .999, *d* = 0.30), dowel (p = .835, *d* = 0.51), or the puddle (p = .987, *d* = 0.22), but older adult participants did modulate their gait speed when crossing different obstacles (Table 3). Specifically, older adults walked more slowly when crossing the branch, curb, or dowel, compared to the puddle (*p* ≤ .001, *d* range: 0.79–1.27), and more slowly over the branch and dowel compared to the curb (*p* < .001, *d* range: 0.40–0.48). There were no differences in crossing speed between the branch and the dowel (*p* > .999, *d* = 0.08).

**Table 3:** Average gait speed during obstructed walking trials in m/s.

	Branch	Curb	Dowel	Puddle
Younger Adults (n = 30)	1.09 ± 0.11	1.14 ± 0.12	1.10 ± 0.12	1.27 ± 0.11
Older Adults (n = 30)	1.02 ± 0.22	1.09 ± 0.23	1.01 ± 0.22	*1.23 ± 0.21

*Note:* *One older adult did not cross the puddle, resulting in a sample size for the puddle of n = 29. m/s = meters per second.

### Measures of lower extremity joint kinematics

Lower extremity joint kinematics from one older adult participant were excluded due to improper thigh marker placement. Although we did not observe an interaction for the lead limb peak ankle dorsiflexion (F(3,176.09)= 0.270, *p* = .847), significant main effects for both group (F(3,59.04)= 5.45, *p* = .023) and obstacle (F(3,176.09)= 97.24, *p* < .001) were present. Older adults crossed the obstacles with greater peak ankle dorsiflexion on the lead limb (*p* = .023, *d* = 0.55) when compared to young adults. Further, participants crossed the puddle with less lead limb dorsiflexion compared to all other obstacles (*p* ≤ .001, *d* range: 1.05–1.40) and crossed the curb with less lead limb dorsiflexion than the dowel (*p* = .001, *d* = 0.35). There were no differences between the branch and the dowel (*p* = .191, *d* = 0.19) or the branch and the curb (*p* = .255, *d* = 0.16).

A significant interaction (F(3,176.06)= 2.98, *p* = .033) revealed older adults cross both the branch (*p* = .042, *d* = 0.86) and the curb (*p* = .039, *d* = 0.88) with more trail limb peak ankle dorsiflexion than younger adults. Differences in trail limb peak ankle dorsiflexion were also observed between all the different obstacle combinations, except the branch and the dowel (*p* > .999, *d* = 0.03). Specifically, the puddle elicited less trail limb peak ankle dorsiflexion than the branch (*p* = .001, *d* = 1.04), the curb (*p* < .001, *d* = 0.60), or the dowel (*p* = .001, *d* = 1.07) and the curb elicited less trail limb peak ankle dorsiflexion than the branch (*p* = .012, *d* = 0.44) or the dowel (*p* = .007, *d* = 0.46).

Significant interactions were observed for both the lead limb peak knee flexion angle (F(3,176.09)= 2.82, *p* = .040) and trail limb peak knee flexion angle (F(3,176.22)= 2.78, *p* = .043). Neither the lead limb peak knee flexion angle nor the trail limb peak knee flexion angle differed significantly between older or younger adults for any of the obstacles (*p* = .396, *p* = .662, respectively). For both limbs, the older adults exhibited the least knee flexion while crossing the puddle compared to all other obstacles (all *p* ≤ .001, all *d* ≥ 3.81) and less peak knee flexion while crossing the curb compared to either the branch or the dowel (all *p* < .001, *d* ≥ 1.06). There were no differences in lead limb peak knee flexion angle or trail limb peak knee flexion angle between the dowel and the branch (*p* = .576, *d* = 0.32, *p* > .999, *d* = 0.25, respectively).

Finally, although neither lead limb peak hip flexion (F(3,175.97)= 1.57, *p* = .198) nor trail limb peak hip flexion (F(3,176.06)= 0.13, *p* = .940) angles displayed significant interactions between age and obstacle, significant main effects for obstacle were observed. Lead limb peak hip flexion (F(3,175.97)= 911.19, *p* < .001) and trail limb peak hip flexion (F(3,176.06)= 228.61, *p* < .001) were significantly different among obstacles. Specifically, for both limbs, the puddle crossings elicited less peak hip flexion than all other obstacles (all *p* ≤ .001, all *d* ≥ 1.13) and the curb required less hip flexion than either the branch (*p* < .001, *d* ≥ 0.43) or the dowel (*p* < .001, *d* ≥ 0.56). The dowel elicited more lead limb peak hip flexion than the branch (*p* = .005, *d* = 0.23), but there were no differences between the dowel and the branch for trail limb peak hip flexion (*p =* .976, *d* = 0.03). Means and standard deviations for all peak lower extremity joint angles of both lead and trail limbs are presented in Table 4.

**Table 4:** Mean ± SD of peak lower extremity joint angles of both lead and trail limbs.

		Peak Hip Flexion		Peak Knee Flexion		Peak Ankle Dorsiflexion	
		Lead Limb	Trail Limb	Lead Limb	Trail Limb	Lead Limb	Trail Limb
Young Adults (n = 30)	Branch	67.0° ± 8.9°	46.1° ± 8.0°	105.5° ± 8.6°	113.6° ± 8.4°	8.8° ± 3.8°	7.0° ± 4.5°
	Curb	62.3° ± 8.0°	41.5° ± 7.3°	95.5° ± 8.6°	100.2° ± 7.0°	7.9° ± 3.8°	4.7° ± 4.5°
	Dowel	68.2° ± 8.5°	45.6° ± 7.5°	107.4° ± 8.6°	114.4° ± 6.7°	9.5° ± 4.1°	7.3° ± 5.0°
	Puddle	38.2° ± 7.1°	32.8° ± 6.1°	64.0° ± 9.8°	65.8° ± 9.9°	3.8° ± 3.5°	3.8° ± 3.9°
Older Adults (n = 29)	Branch	65.2° ± 12.5°	45.6° ± 8.5°	104.4° ± 10.9°	114.1° ± 6.5°	10.8° ± 4.3°	11.3° ± 5.9°
	Curb	61.8° ± 10.5°	41.4° ± 8.2°	94.5° ± 9.7°	99.1° ± 7.6°	10.4° ± 4.2°	9.1° ± 5.8°
	Dowel	68.3° ± 11.7°	45.6° ± 8.9°	107.4° ± 8.2°	116.1° ± 9.3°	11.5° ± 4.5°	11.4° ± 6.1°
	Puddle	38.5° ± 7.6°	33.0° ± 5.8°	58.6° ± 11.5°	61.8° ± 9.9°	6.2° ± 4.3°	6.2° ± 4.8°

*Note:* Joint kinematic data was unavailable for one older adult and one other older adult did not cross the puddle, resulting in a sample size for the puddle of n = 28.

## Discussion

The purpose of this study was to compare measures of obstacle crossing behavior between young and older adults using four real-world or simulated real-world obstacles: a branch, a parking curb, a dowel rod, and a puddle. In support of our hypothesis, older adults did exhibit higher toe clearance in both leading and trailing limbs during crossing as compared to younger adults, however, there was no difference between groups for approach or landing distance. The increased toe clearance was likely driven by the greater peak ankle dorsiflexion on both the lead and trail limbs, as peak knee and hip measures did not differ between young and older adults. Overall, our results are consistent with previous literature showing older individuals increase foot clearance in both the leading and trailing limbs to prevent tripping, supporting the idea that the obstacles were perceived as a greater risk by older individuals when compared to our younger adults [12]. By increasing toe clearance of both limbs, older individuals exhibit a greater margin of safety, decreasing the likelihood of contacting the obstacle and potentially falling. We further hypothesized that in older adults, the dowel would elicit increased measures of obstacle clearance (increased approach and landing distance, as well as increased toe clearance and decreased crossing velocity) when compared to the other obstacles. While older adults did cross the dowel with greater toe clearance than all other obstacles, our previous investigations found that young adults exhibit the same response [20], suggesting this is not an adaptation associated with aging, but may be a response to perceptions of the dowel obstacle’s foreignness and fragility.

Contrary to our hypothesis, the lack of differences observed between groups for approach and landing distance can be likely be attributed to the similarities in gait speed and step length between groups at baseline. Neither gait speed nor step length differed between young and older adults during unobstructed, baseline walking. Likewise, when comparing crossing speeds, although a previous study found older individuals decreased their speed more than younger individuals when crossing over a dowel obstacle [35], we observed no differences in crossing speed between older and younger individuals. The participants in our study reported being healthy, both groups walked with similar preferred walking paces during the familiarization trials (Table 1), and SPPB scores indicate that both the young and older adults were unimpaired and did not demonstrate marked functional deficits. In fact, among older adults in this study, the crossing speed of the puddle and the unobstructed, self-selected walking speed were similar (i.e., 1.23 ± 0.21 m/s vs. 1.25 ± 0.17 m/s). This may indicate that the puddle did not impose a meaningful “threat”, and participants did not require gait modulation. However, obstacle crossing speeds were the slowest over the branch and dowel indicating a possible greater perceived threat. The sample in the aforementioned study was slightly older than our older adult sample (71 ± 5 versus 69 ± 6 years old in our study) and reported some participants with osteoarthritis, vision impairments and musculoskeletal weakness, so greater aging deficits were likely observed in their study [35]. In relatively older populations, it is likely that results are magnified, and balance deficits are more pronounced than in our sample.

In support of our second hypothesis, that the dowel would elicit increased obstacle clearance compared to the other obstacles, our results indicate the dowel did provoke the greatest lead and trailing limb toe clearance compared to all other obstacles. We posit that, compared to the more common, everyday obstacles, the foreignness of the dowel may play a role in the greater toe clearance, specifically considering the similarities in height, width, and composition to the branch. We speculate that when people encounter new obstacles, greater caution is taken to ensure crossing success. Additionally, the dowel and the branch are not solid, block-type obstacles like the parking curb. Previous literature suggests solid obstacles may provide more insight into the height and depth of the obstacle itself, allowing for greater perception of the obstacle location in space [10]. With a better understanding of where the obstacle was in space, participants may have felt more comfortable getting closer to the solid obstacles than the obstacles in which perceptions in space may have been more difficult (i.e., the dowel and the branch).

Indeed, the branch elicited the second greatest toe clearance of all obstacles, which may indicate clearance was a product of obstacle dimensions (i.e., height) or perceptions of fragility [36]. Work by Patla and colleagues [36], highlight that past experiences inform how we cross obstacles and that humans tend to adapt a more cautious crossing strategy for obstacles perceived as fragile [36], which can be seen in our study with greater toe clearance above both the dowel and the branch. Additionally, the dowel and the branch promoted longer approach and landing distances than the curb and puddle, although the dowel and branch did not differ from each other. While there are similarities between lab-based and real-world obstacles of similar dimensions, as evidenced by approach and landing distance for the dowel and the branch, the differences between the same obstacles in vertical toe clearance is intriguing. Particularly because, although the dowel and branch obstacles are similar in physical dimension (e.g., height, shape, fragility), the lack of difference in the approach distance, landing distance, and crossing step length between the two obstacles suggest that the endpoints of the crossing steps are the same. Despite this, older adults exhibit exaggerated vertical toe clearance while crossing the dowel compared to the branch indicating the shape of the toe trajectory differs, regardless of the similar endpoints.

The puddle and curb were the only obstacles with substantial depth (300 mm and 215 mm, respectively), which likely explains the shorter approach and landing distances, as well as the longest crossing step length for the puddle. When encountering obstacles with more depth, such as bags or boxes, there may be an increased risk of falling due to the lack of change in step length [37]. Real-world obstacles vary in dimension, increasing the likelihood of falling if the crossing step is not adjusted to account for an obstacle’s shape. When accounting for the depth of the curb and the puddle, the crossing step length becomes more similar for all the obstacles, indicating that the decreased approach and landing distances may be an artefact of obstacle depth [20]. Considering it is vital to adjust step length to avoid an obstacle, a lack of adjustment could indicate an increased risk of obstacle contact and a subsequent fall.

Similarly, the ability to cross an obstacle with either leg as the lead limb has benefits. Crossing with the non-dominant leg allows for increased stability in the first half of obstacle crossing because the dominant leg is stable on the ground. Conversely, crossing with the dominant leg allows stability when the trailing leg is crossing the obstacle because the dominant leg is stable on the ground for the final half of crossing. Since the majority of obstacle collisions occur with the trailing leg [29,38], increased stability during the second half of obstacle crossing may be more useful for avoiding contacts [6]. Participants in our study successfully crossed the obstacles while leading with both the dominant and non-dominant legs, even with consistent individual start points. Collectively, this demonstrates both age groups could alter leading limb selection smoothly and cross obstacles to maintain a regular gait pattern rather than dramatically adjusting step length, which could lead to instability and unsafe obstacle crossing. Individuals in our study demonstrated good balance, indicated by SPPB scores, and switched leading limbs freely without compromising perceptions of crossing safety, evidenced by safety ratings from the older adults. Likewise, decreased crossing velocity for the branch, curb and dowel indicates participants modulated gait speed, rather than crossing limb, when confronted with erect obstacles.

With regard to lower body joint kinematics, participants crossed the puddle comparably to unobstructed normative walking data at a self-selected pace [39], with significantly lower peak joint angles compared to all other obstacles. The dowel and branch elicited the most hip and knee flexion, indicating participants adapted a more flexible approach to successful crossing, likely due to the added height of these obstacles. Of note, all peak joint kinematics were still less than what is required for typical activities of daily living such as putting on pants or tying shoes [40]. We speculate that less mobile older adults would adapt different crossing strategies than presented in this study, especially if they do not meet minimum range of motion criteria for activities of daily living, such as stair climbing [40,41].

One potential limitation of this study is the different obstacles that were selected for use; we selected a variety of obstacles with different dimensions, textures, and visual characteristics in an effort to improve the external validity of the findings. Future investigations should include varied obstacle types, with consistent dimensions to compare the impact of obstacle characteristics on obstacle negotiation strategies. Although this study used obstacles often found in the real world, this investigation is limited by the reliance on a 3D motion capture system confined to a laboratory setting. Future investigations should consider evaluating obstacle crossing of real-world obstacles in a natural environment. Further, our investigation employed a toe marker positioned at the head of the 2^nd^ metatarsal which overestimates foot clearance and approach distance, inflating the magnitude of these measures. Caution should be used when comparing to this data or extrapolating these values for fall risk. Finally, as mentioned above, our sample demonstrated good functional abilities, which likely contributed to the lack of significant differences between groups.

## Conclusions

In conclusion, this study compared measures of obstacle crossing behavior between young and older adults using four real-world or simulated real-world obstacles: a branch, a parking curb, a dowel rod, and a puddle. Overall, older adults cross obstacles with increased vertical toe clearance and ankle dorsiflexion when compared to young adults. In contrast, older and younger adults demonstrated similar horizontal clearance, crossing step length, and obstacle crossing speeds. Collectively, these results better our understanding of how realistic obstacle crossing changes with age and support the notion that obstacle clearance is a priority during safe obstacle crossings for older adults.

Although the dowel promoted the greatest vertical margin of safety in toe clearance when compared to all other obstacles, the similarities in obstacle crossing measures between the dowel and the branch suggest obstacles with approximate dimensions may elicit interchangeable crossing strategies. Obstacle depth influences crossing strategy [42] and our curb and puddle, with substantial depth, provoked smaller approach and landing distances; participants favored the conservation of step length rather than a larger margin of safety when crossing these obstacles. By using the traditional dowel in obstacle crossing studies, generalizability of obstacle clearance measures may be limited when compared to more realistic multi-dimensional obstacles. Evaluating real-world obstacles, beyond the traditional dowel rod, highlights more realistic strategies that can inform future fall prevention strategies to decrease the occurrence of real-world falls in older adults.

## Supporting information

S1 Dataset Supplementary obstacle crossing data from young and older adults. (XLSX)
